# Spatiotemporal segmentation of contraction waves in the extra-embryonic membranes of the red flour beetle

**DOI:** 10.1186/s12859-025-06259-1

**Published:** 2025-10-21

**Authors:** Marc Pereyra, Mariia Golden, Zoë Lange, Artemiy Golden, Frederic Strobl, Ernst H. K. Stelzer, Franziska Matthäus

**Affiliations:** 1 Goethe University Frankfurt, Frankfurt Institute for Advanced Studies, Ruth-Moufang-Straße 1, Frankfurt AM, 60438 Hessen Germany; 2 Goethe University Frankfurt, Buchmann Institute for Molecular Life Sciences, Max von Laue Str. 15, Frankfurt AM, 60438 Hessen Germany

**Keywords:** Movement segmentation, Particle image velocimetry (PIV), Tissue cartography, Image analysis, Tissue contraction, Extra-embryonic development, *Tribolium castaneum*

## Abstract

**Background:**

In this paper, we introduce an image analysis approach for spatiotemporal segmentation, quantification, and visualization of movement or contraction patterns in 2D+t and 3D+t microscopy recordings of biological tissues. The development of this pipeline was motivated by the observation of contraction waves in the extra-embryonic membranes of the red flour beetle *Tribolium castaneum*. These contraction waves are a novel finding, whose origin and function are not yet understood. The objective of the proposed approach is to analyze the dynamics of the extra-embryonic membranes in order to provide quantitative evidence for the existence of contraction waves during late stages of embryonic development.

**Results:**

We apply the pipeline to live-imaging data of *Tribolium* embryonic development recorded with light-sheet fluorescence microscopy. The proposed pipeline integrates particle image velocimetry (PIV) for quantitative movement analysis, surface detection, tissue cartography, and algorithmic identification of characteristic movement dynamics. We demonstrate that our approach reliably and efficiently detects contraction waves in both 2D+t and 3D+t recordings and enables automated quantitative analyses, such as measuring the area involved in contractile behavior, wave duration and frequency, spatiotemporal location of the contractile regions, and their relation to the underlying velocity distribution.

**Conclusions:**

The pipeline will be employed in future work to conduct a large-scale characterization and quantification of contraction wave behavior in *Tribolium* development and can be readily adapted for the identification and segmentation of characteristic tissue dynamics in other biological systems.

## Background

We present a method to systematically detect and analyze a recently observed but yet undescribed phenomenon of contraction waves in the extra-embryonic (EE) membranes during the embryonic development of the red flour beetle *Tribolium castaneum*. The method can generally be applied to analyze movement patterns in biological tissues characterized by bidirectional motion, given in the form of 2D+t or 3D+t microscopy time lapse sequences.

Cell contractions and wave-like tissue movements—which often constitute major milestones in the evolution of species [1–3]—play important roles during embryonic development (reviewed in: [4, 5]). In particular, apical contractions of epithelial cells are crucial in morphogenesis [6, 7]. Tissue movement and contraction waves in developmental biology involve complex interactions between mechanical forces and cellular signaling [8, 9]. Contractile forces generated by the actin-myosin cytoskeleton are critical for morphogenesis, with different modes of actomyosin contraction producing various temporal and spatial patterns of force generation [10].

Cell contractions have been observed during dorsal closure in the embryogenesis of the most commonly studied insect model organism, the fruit fly *Drosophila melanogaster*. Here, cells of the amnioserosa, its sole EE membrane that covers only a small dorsal area on top of the yolk [11, 12], apically contract. This leads to a reduction of EE tissue area, which is important for subsequent internalization [13–15]. In contrast to *Drosophila*, *Tribolium* exhibits two separate EE membranes: the serosa, which envelopes the entire embryo proper and the yolk, and the amnion, which covers the embryo proper ventrally. The EE membranes eventually rupture in the anteroventral region and rapidly withdraw to the dorsal side (Additional video 1). This process signals the onset of dorsal closure [16], which happens roughly one day before hatching. With its EE membrane architecture and dynamics, *Tribolium* can be considered representative of the embryonic development of the most numerous order of insects, the Coleoptera [17].

Recent studies have advanced our understanding of tissue movement and contraction waves in developmental biology through computational and imaging approaches, where motion analyses and segmentation play crucial roles. For instance, Aleksandrova et al. use movement analysis in the form of particle tracking, particle image velocimetry (PIV) and image registration to distinguish between local active cellular motion and large-scale tissue movements during organogenesis [18]. Tracking and PIV are the classical methods to quantify tissue movement in biological samples, in particular also in the study of developmental processes [19–22]. Segmentation, on the other hand, is a common method to identify objects based on the local intensity patterns in the image. In the analysis of biological tissues, it is often used to quantify cell size and morphology. Examples are the work of Capitán-Agudo et al. who have optimized 3D segmentation to capture the 3D cell shape in curved epithelial tissues in *Drosophila* [23], or the work of Xie and Martin, who have used boundary-based segmentation and an alignment of cell area with myosin pulses to identify and classify contractile pulses in epithelial cells during *Drosophila* ventral furrow invagination [24]. Segmentation has also been used to study cell contraction and intercalation processes during *Tribolium* development [25]. Many efficient software packages exist that support segmentation of cells from microscopy images of biological tissues, for instance Ilastik [26], Cellpose [27] or CartoCell [28].

Our approach differs from these works as we do not use static image intensity, but the motion analysis itself for segmentation of tissue areas showing specific behavior. A similar strategy was chosen by Yao et al. to count insects on a glass plate [29]. Our approach is applied to the investigation of EE membrane dynamics in *Tribolium*.

We analyzed fluorescence live imaging data of *Tribolium* embryos expressing either a nuclear label or a membrane label, recorded using light sheet fluorescence microscopy (LSFM). The proposed image analysis approach for this data consists of three main components that address different aspects of our specific problem. One key feature stems from the geometry of the EE membranes in *Tribolium* embryos. The outer EE membrane, the serosa, is a continuous epithelial layer that envelopes the whole embryo from gastrulation to rupture, which can be geometrically described as a closed surface in 3D space. A powerful approach for analyzing such surfaces is through extracting surface masks and projecting the intensities onto 2D maps [30]. We include this approach in our workflow, as it has the additional advantage of separating the EE membrane movements from those of the embryo proper.

The second pillar of our approach is ensuring the robust and efficient quantification of tissue dynamics based on 2D image and 3D volumetric microscopy data. We use PIV, which is a common image analysis technique to quantify the movement of structures between consecutive time points. PIV is well-suited for collective cell motion and can be applied to data where cell segmentation is hard or impossible [31–33]. In particular, we use the quickPIV software package, which is an efficient and open-source PIV implementation handling 2D and 3D images. QuickPIV also includes features that are used here, such as masked PIV and normalized cross-correlation using squared differences, which is more robust than the commonly used normalized cross-correlation [34].

Lastly, our approach proposes an original algorithm for segmenting the characteristic back-and-forth motion that we observe during contraction waves in the EE membranes of the *Tribolium* embryo. We make use of the observation that the contraction waves are characterized by alternating back-and-forth movements of large patches of EE membrane cells in *Tribolium*. They seem to start in the anterodorsal area and propagate in the posteroventral direction. This movement repeats multiple times before the rupture and retraction of EE membranes, which happens at the late stage of embryonic development (Additional video 1). Our algorithm uses the relative changes in the movement direction, given by the PIV vectors, to detect the specific motion pattern of the contraction waves. The algorithm requires only a few parameters that intuitively shape the range of detectable patterns.

In the following, we introduce the methods that were used to analyze EE membrane dynamics and segment contraction waves. We developed and used methods for both 2D and 3D data and applied them to three different representations of our time-lapse data: (1) 2D maximum intensity projections of a lateral view of the embryo, (2) fused 3D volumes from multi-view LSFM recordings, and (3) cylinder projections based on the surface mask where the EE membranes get unrolled and mapped to 2D.

## Methods

### Tribolium castaneum transgenic lines and husbandry

This study used two different transgenic *Tribolium* lines created using the ACOS vector concept [35]. The (mCe/mCe) homozygous ACOS{ATub’H2A/H2B°NB-mRuby} #1 subline expresses mRuby2-labeled anti-H2A/H2B nanobodies (nuclear label) under control of the alpha-tubulin promoter [36]. The (mCe/mCe) homozygous ACOS{HbB:DSCP’#O(MEME)-tdTomato} #1 expresses a tdTomato-labeled GAP43 membrane anchor tag [25] (membrane label) with an extended linker sequence under control of a HbB enhancer/DSCP promoter combination [37]. Detailed data on the lines are available on request. Beetle cultures were maintained under standard conditions at 32 °C, ~70% relative humidity [35]. For embryo collection, beetles were transferred to fresh egg-laying medium, and the egg laying culture was incubated for 1 h at room temperature. After collection, embryos were further incubated for 45 h at 32 °C. Dechorionation was performed during the second day of incubation.

### Image acquisition

Live imaging data of *Tribolium* embryonic development were acquired using two different custom digital scanned laser light sheet fluorescence microscopes [38, 39]: the embryo from the ACOS{ATub’H2A/H2B°NB-mRuby} #1 subline (DS0001, DS0003-5) was recorded using a single-illumination setup employing a 2.5 × 0.06 NA illumination objective (422320-9900-000, Carl Zeiss), a 10 × 0.3 NA detection objective (420947-9900-000, Carl Zeiss), and a 14-bit high-resolution CCD camera (Clara, Andor). The detection objective/camera combination resulted in a 0.645 µm lateral pixel pitch. Illumination was performed with a 561 nm laser line (Cobolt Jive CW 561, Omicron Laserprodukte GmbH) with a nominal laser power of 1.8 mW and an exposure time of 50 ms per plane. Fluorescence was detected through a 607/70 single-band bandpass filter (FF01-607/70-25, Semrock/AHF Analysentechnik AG). Sample preparation, mounting and microscope calibration were performed as previously described in [40]. Below the embryo, an agarose column containing 1 μm fluorescent beads (T7282, Thermo Fisher Scientific) was placed that serves as fiducial landmarks to aid registration. Planes were recorded with a spacing of 2.58 μm. One embryo (DS0001) was imaged along four views with rotation steps of 90° and another three embryos were imaged along one view, all with a temporal interval of 3 min.

The embryos from the ACOS{HbB:DSCP’#O(MEME)-tdTomato} #1 subline (DS0002, DS0005-9) were recorded using a double-illumination setup employing two 2.5 × 0.06 NA illumination objective (422320-9900-000, Carl Zeiss), a detection objective 10 × 0.3 NA (420947-9900-000, Carl Zeiss), and a 12-bit CMOS camera (DMK 33UX174, The Imaging Source). The detection objective/camera combination resulted in a 0.586 μm lateral pixel pitch. Illumination was performed with a 561 nm laser line (TOPTICA iChrome CLE) with a final laser power of 689 μW and an exposure time of 8 ms per plane. Fluorescence was detected through a 607/70 single-band bandpass filter (AHF, Semrock, Brightline HC 607/70, F39-608). Embryos were mounted in a column of 1% (w/v) low-melt agarose as previously described in [41]. Additionally, a small volume of agarose containing 1 μm fluorescent beads (T7282, Thermo Fisher Scientific) was sucked into the capillary right underneath the embryo in order to occupy one field of view with the embryo. Planes were recorded with a spacing of 2.34 μm. One embryo (DS0002) was imaged along six views with rotation steps of 60° and another four embryos were imaged along one view, all with a temporal interval of 3 min.

### Data processing

#### Generation of fused 3D volumes

For the multi-view datasets (DS0001 and DS0002), each frame—where "frame" refers to the time point in the recording—had its z-stacks registered and fused with the help of the BigStitcher Fiji plugin [42]. Affine registration was done for each time point using only fluorescent beads as interest points for the nuclear-labeled dataset and using bright maxima of the outer membranes of the embryo as interest points to further refine the registration for the membrane-labeled dataset.

For the dataset deriving from the nuclear-labeled line recorded along four views (DS0001), the signal from the fluorescent beads was used for extracting the point spread function (PSF) for deconvolution. Five iterations of deconvolution were done using BigStitcher’s "Efficient Bayesian, Optimisation I" algorithm. This produced high-quality 3D+t dataset covering the late embryonic development of *Tribolium*. For the membrane-labeled dataset (DS0002), due to a better registration based on the interest points from the sample, deconvolution was not needed to get good quality cartography maps and cell segmentations.

#### Lateral maximum intensity projections

Based on the fused data we generated 2D+t datasets consisting of maximum intensity projections from one of the lateral halves of the embryos, where the contraction waves are clearly visible. For the case where double-illumination data were available, related images were combined by calculating their average.

#### Surface mesh

Until rupture the serosa constitutes the surface of the embryo. Therefore, we included a data-processing step in our pipeline for extracting only the surface of the embryo, allowing us to restrict our analyses to the EE membrane signal. The surface detection for each volume was implemented as follows: First we generated a binary segmentation of each fused 3D volume into foreground (*Tribolium* signal) and background. The boundary of this segmentation does not correspond directly to the surface of the embryo. This is because the foreground segmentation is based on either the nuclei signal, which is inherently sparse, or the membrane signal, which is usually faint and may contain gaps. From here, we sampled a 3D point cloud from the segmentation and computed their 3D alpha shape with pymeshlab [43]. This generates a surface mesh that connects points in the segmentation, allowing us to interpolate any gaps along the surface. Lastly, we removed facets in the interior of the alpha shape, as these do not correspond to the surface. The surface meshes were used for restricting the PIV analyses to the EE membranes, and also for tissue cartography, which is covered in the next section.

#### Cylinder projections

To reduce dimensionality and allow analysis with 2D methods, we followed the work of Streichan et al. [30] and used their freely available "Image Surface Analysis Environment" (ImSAnE) software. This allowed us to generate an atlas of overlapping maps, in which we analyzed the surface dynamics of the EE membranes on cylinder projections. Using ImSAnE, we extracted the voxels of interest from the 3D volume using the surface mesh (Sect.  ''Surface mesh'') and projected the voxel intensities to their respective pixels in a flat 2D geometry. We included 9 radial layers of the mesh, 6 towards the inside and 3 towards the outside, to capture all nuclei of the EE membranes without including signal from the embryo proper inside. This gave us a stack of 10 2D cylinder maps from which we created a maximum intensity projection. We represented the information on up to two cylinder projections with a 180° rotational offset to each other along the main axis of the mesh. We used the respective maximum intensity cylinder projections to create 2D+t time-lapse videos for 2D PIV.

We needed to make a few considerations when computing PIV on cylinder projections, especially when representing the results on 2D maps. In cylinder projections, both distance and angle distortions are introduced. They increase gradually towards the polar regions from the equator, which has no distortion. We corrected for length distortions introduced by cartography to ensure that elongated or shortened PIV vectors do not impact our wave segmentation. We used a uniform sampling with 10 pixels distance to create a 2D grid. Using the function *properLength* in ImSAnE we calculated the distance that the sample points would have in 3D space. We obtained two values for each 10 × 10 tile of the grid: longitudinal distortion (from pole to pole) which is characterized by shortening towards the poles, and latitudinal distortion (parallel to the equator), which is characterized by elongation towards the poles. We then multiplied the distortion fields with the 2D PIV vector field to obtain corrected vector lengths. We did not correct for angle distortions because these only become significant ($\gg$10°) very close to the poles. Instead, we crop the polar regions before analysis.

### Analyzing EE membrane dynamics with PIV

We used quickPIV [34] to quantify tissue motion in 2D and 3D. In the case of the 3D data, we restricted the PIV analyses to the EE membranes by providing a 3D mask of the surface of the embryo. This speeds up the computation and results in PIV vector fields that predominantly capture EE membranes dynamics, reducing contributions from the movement of the subjacent embryo proper. The 3D surface mask was generated by combining surface masks derived from surface meshes of the embryo at different time points (Sect.  ''Surface mesh''). We used the same mask for all 3D PIV analyses to ensure that all results share the same set of coordinates.

We computed PIV between each pair of consecutive frames for the entire 3D+t microscopy data, as well as 2D+t projections, resulting in 3D+t and 2D+t vector fields, respectively. In the rest of the paper, we will refer to an arbitrary vector within the PIV results by its spatial, $\textbf{p} = (x,\;y)$ or $\textbf{p} = (x,\;y,\;z)$, and temporal, *t*, coordinates: ${\textbf{v}}_{\textbf{p},\;t}$. The wave segmentation algorithm revolves around analyzing the change in direction of individual PIV vectors over time. In other words, the input to the algorithm is the set of PIV vectors at the spatial coordinate $\textbf{p}$ across all frames, which we denote as the vector time series ${\textbf{v}}_{\textbf{p},\;{t}}$.

### Temporal segmentation of characteristic dynamics based on PIV vector fields

**Fig. 1:**
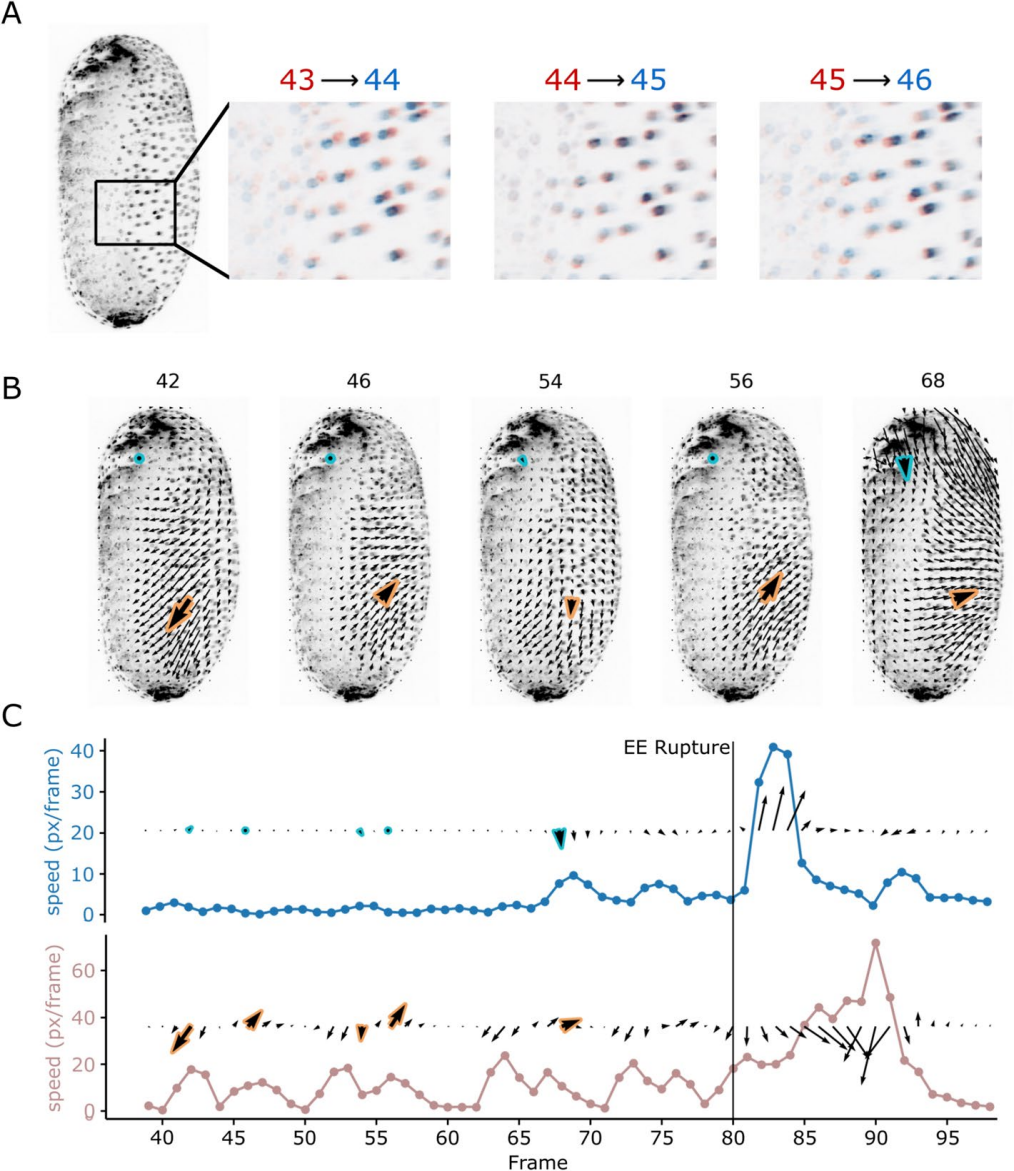
Quantification of contraction waves through PIV. **A** Lateral view of *Tribolium castaneum* (left). Contraction waves are visible from the lateral views as dorsoventral back-and-forth movements of cells of the EE membranes. The three panels on the right illustrate the movement of the EE membrane nuclei by overlying the intensities of two consecutive frames: red for the first frame and blue for the second frame. **B** 2D+t PIV results on lateral maximum projections. The contraction waves are captured in the PIV vector fields and are spatially coherent. Two positions of the vector field are highlighted: the blue-bordered vectors, and the orange-bordered vectors. **C** Temporal evolution of the PIV vectors at the highlighted positions in B. The blue vectors do not show any contraction waves, but still show sporadic velocity peaks of about 7–10 px/frame before rupture, as well as a large velocity peak after rupture. The orange vectors illustrate the appearance of PIV vectors in regions of contraction waves. In particular, this vector series shows five waves before rupture and at least one wave after rupture. There is a high-velocity peak around frame 90 due to the retracting EE membranes crossing this position of the vector field. Analysis was performed on DS0001

We developed the wave segmentation algorithm around the *a priori* observation that contraction waves in the EE membranes of *Tribolium* are characterized by two consecutive phases of tissue movement in different directions: an initial phase of posteroventral movement over several frames, followed by a phase of movement with a strong dorsal component for several frames, see Fig. 1A and B. We refer to these two phases as the V and D phases, respectively, highlighting the importance of the ventral and dorsal components of the movements in the contraction waves. Pairs of consecutive V and D phases can be found in the PIV vector fields by identifying instances where the direction of the vector fields shifts from ventral to dorsal over successive time points. This must be implemented with the most generality, allowing for arbitrary angles between the V and D phases to account for the inherent variability of biological processes. Following these considerations, we constructed our algorithm around a set of flexible conditions defining a range of detectable motion patterns. For each vector time series, ${\textbf{v}}_{\textbf{p},\;{t}}$, the algorithm proceeds as follows:

1. We first adopt a sliding window approach to detect frames of locally maximum and minimum similarity with a reference direction. In our case, this direction points towards the ventral line of the embryo, since the V phases mark the start of the contraction waves.
2. Secondly, we subject each pair of consecutive local maxima and minima from the previous step to a set of angle-based criteria to determine whether they correspond to the V and D phases of a contraction wave. If these criteria are satisfied, we proceed to generate temporal segmentations of the waves.
3. After independently generating a temporal wave segmentation for each spatial position of the PIV vector field, we apply post-processing steps to refine the spatial segmentation of the tissue involved in the contraction wave.

#### Detection of V phase and D phase candidates

Our approach for segmenting the contraction waves starts by first identifying candidate locations of the V and D phases, which we further process and aggregate to obtain valid wave segmentations. This initial step of finding candidate locations is based purely on directional information. More precisely, we search in the PIV vector time series ${\textbf{v}}_{\textbf{p},\;{t}}$ for a pattern of *N* consecutive vectors pointing coherently in one direction, followed by *M* vectors pointing coherently in a different direction. In the case of contraction waves in *Tribolium*, the direction of the first phase (the V phase) is constrained to the posterioventral direction. This a-priori constraint on the direction of the V phases is implemented in our algorithm by specifying a reference direction. For example, to analyze the 2D lateral projections of DS0001, we choose the normalized reference vector, $\textbf{r}$, pointing along the angle bisector of the third quadrant (225°, see Fig. 3A). In addition to the reference vector, which establishes the average direction of the V phases, we further define a broader region of allowable directions by considering an angular deviation, $\theta _{\textbf{r}}$, around $\textbf{r}$ (illustrated with $\theta _{\textbf{r}}$ = 40° in Fig. 3A). The range of possible directions of the D phase is more variable: it is broadly constrained within 180° of the dorsal direction. In addition, we impose a minimum angle between the average directions of the V and D phases ($\theta _{\textrm{VD}}$ in Fig. 3A).

This pattern can be detected by a sliding window approach on the normalized PIV vector field. For every frame *t* we compute the dot product between the average vector of the normalized PIV vectors around *t*, [$t-N,\;t+N$], and the normalized reference vector for the V phase, $\textbf{r}$. Due to the sliding window approach this dot product is a function of time, denoted as $\rho (t)$ (Fig. 3B). This measure is maximized if all vectors in the sliding window point in the same direction as the reference vector. In other words, $\rho (t)$ is maximized when the vectors within [$t-N,\;t+N$] display low intra-group variability and simultaneously point in the direction of $\textbf{r}$. On the other hand, $\rho (t)$ is minimized around time points where the PIV vectors in the sliding window point collectively away from $\textbf{r}$.

Most notably, $\rho (t)$ provides an approximate location of the centers of the V and D phases of the waves. The local maxima of $\rho (t)$ correspond to time points of coherent movement in a similar direction to $\textbf{r}$, which correlates with the V phases. On the other hand, the local minima of $\rho (t)$ mostly indicate frames where PIV vectors are coherently pointing in a different direction to $\rho (t)$, corresponding to the location of the D phases. Local minima can also indicate incoherent movement away from $\rho (t)$. Thus, we consider each pair of consecutive local maxima and minima as potential landmarks for contraction waves, as illustrated in Fig. 3B.

The normalized reference vector $\textbf{r}$ plays a major role in the wave segmentation algorithm, and it is important to adjust the direction of $\textbf{r}$ to each dataset. In addition, some data representations require different reference vectors for different positions in the PIV vector field, $\textbf{r}_\textbf{p}$. This is the case for our 3D+t datasets and our 2D+t cylinder projections, as the ventral direction at each location is determined by the geometry of the surface of the embryo.

#### Extracting wave segmentations

This step takes each pair of consecutive local maxima and minima ($t_{\textrm{max}}$ and $t_{\textrm{min}}$) extracted from $\rho (t)$ and evaluates whether the PIV vectors surrounding these time points fit to the expected pattern of the movement characteristic of the contraction waves. If this is the case, a segmentation of the V and D phases is generated. Specifically, we evaluate the following set of conditions in the order presented below, where failing any step results in the unsuccessful segmentation of a wave:

1. We first check whether the PIV vector at $t_{\textrm{max}}$ (${\textbf{v}}_{\textbf{p}, t_{\textrm{max}}}$) points in the reference direction, $\textbf{r}$. The vector at this frame is characterized by low intra-group angle variances, making it representative of the movement in the V phase. This is tested with a threshold on the normalized dot product: $\hat{{\textbf{v}}}_{\textbf{p},\;t_{\textrm{max}}} \cdot \textbf{r} > \theta _{\textbf{r}}$ (Fig. 3D left).
2. We then check whether the angle between the representative vectors of the V and D phases is noticeable. The representative vector for the D phase is obtained from $t_{\textrm{min}}$, ${\textbf{v}}_{\textbf{p},\;t_{\textrm{min}}}$. This is tested with a threshold on the following normalized dot product: $\hat{{\textbf{v}}}_{\textbf{p},\;t_{\textrm{max}}} \cdot \hat{{\textbf{v}}}_{\textbf{p},\;t_{\textrm{min}}} < \theta _{\textrm{VD}}$ (Fig. 3D left).
3. We proceed by creating a segmentation of the V phase by the process described below (illustrated in Fig. 3D middle panel). The segmentation *V* is a set initialized to contain a single frame, $V = \{ t_{\textrm{max}},\;\ldots\}$. We expand this segmentation by iterating over the adjacent frames starting at $t_{\textrm{max}}$ from nearest to farthest, and deciding whether to add them to the set *V*. A frame *t* is added to *V* if two conditions are met:
  - The vector at *t* points in a similar direction as the current segmentation of the V phase. Since the segmentation *V* may contain multiple vectors, this is computed with the dot product with the average vector in *V*: $\hat{\textbf{v}}_{\textbf{p},\;t} \cdot \textbf{V}_{\textrm{avg}} > \theta _{\textrm{V}}$.
  - The vector’s magnitude is sufficiently large: $||\textbf{v}_{\textbf{p},\;t}|| > M_{\textrm{min}}$.
  The same process is applied to generate the segmentation of the D phase, with the only difference of using $\theta _{\textrm{D}}$ instead of $\theta _{\textrm{V}}$. In some rare cases the V and D segmentations are not directly adjacent, for instance when the vectors in the interface exhibit a very low magnitude. If this occurs, the vectors at the interface are added to the phase with the most similar direction.
4. We impose additional conditions on the segmented V and D phases. In particular, we discard both the V and D segmentations if the average speed of the V phase is smaller than a threshold, $M_{\textrm{avg}}$. In addition, we include a threshold on the total distance traveled (total displacement) during a wave, which is computed as the sum of magnitudes for the duration of the V and D phases of a contraction wave.

At the end of the segmentation step, we obtain V and D segmentations for each contraction wave with low intra-angle variance (parameterized by $\theta _{\textrm{V}}$ and $\theta _{\textrm{D}}$) and significant inter-angle variance (determined by $\theta _{\textrm{VD}}$). Speed information is used to confine the segmentation to regions with sufficient movement. The parameters used to segment contraction waves in the different dataset representations are provided in Table 1.

**Table 1:** Parameters for wave segmentation algorithm

	Lateral projections	3D fused volumes	Cylinder projections
$r_x$	$-\cos (45^{\circ })$	$-\sin (8.4^{\circ })$	$\pm \cos (45^{\circ })$
$r_y$	$-\sin (45^{\circ })$	$\sin (45^{\circ })\cos (8.4^{\circ })$	$-\sin (45^{\circ })$
$r_z$	0	$-\cos (45^{\circ })\cos (8.4^{\circ })$	0
$\theta _{\textbf{r}}$	$40^{\circ }$	$50^{\circ }$	$50^{\circ }$
$\theta _{\textrm{V}}$	$60^{\circ }$	$60^{\circ }$	$40^{\circ }$
$\theta _{\textrm{D}}$	$60^{\circ }$	$60^{\circ }$	$40^{\circ }$
$\theta _{\textrm{VD}}$	$30^{\circ }$	$30^{\circ }$	$30^{\circ }$
$M_{\textrm{min}}$	0.5 px/frame	0.5 vx/frame	1.0 px/frame
$M_{\textrm{avg}}$	1.0 px/frame	1.0 vx/frame	3.0 px/frame
$M_{\textrm{total}}$	10 pixels	10 voxels	10 pixels

#### Spatial post-processing of the wave segmentation

The temporal wave segmentation algorithm is independently applied to the vector time series at each spatial coordinate, ${\textbf{v}}_{\textbf{p},\;{t}}$, and therefore does not include any operations to ensure that wave segmentations are spatially coherent. However, several factors throughout the pipeline impose spatial coherence of the wave segmentation results. Firstly, the phenomenon of interest concerns collective tissue motion, which is inherently spatially coherent. Secondly, spatial coherence is increased as a side product of PIV post-processing, which is commonly applied to remove high-frequency noise and vector artifacts.

However, we also obtained spurious segmentations in other regions of the embryo, either from small wave-like movements in EE membranes or from twitching movements of the embryo proper. We remove small wave-like detections with morphological operations. Namely, we perform morphological opening to the V and D segmentations to remove thin segmentations, followed by high-pass filtering by the size of the connected components in the segmented phases. Artifactual segmentations due to movements in the embryo proper start after rupture, as EE retraction proceeds and the embryo relocates itself within the egg. We remove these by filtering segmentations whose centroid is near the head of the embryo.

### Visualization

#### Kymographs

Kymographs are a commonly used method for visualizing spatiotemporal (2D+t) datasets as a 2D plot where one of the axes is the temporal dimension. The other axis of the plot can correspond to a line in the spatial dimension, or a projection of spatial data onto this line. We here used summed intensity projections of the data along one of the spatial axes, either X or Y, for all kymographs. We use kymographs to show the wave segmentation results across time for the 2D+t dataset of maximum intensity projections, Fig. 3, as well as the 2D+t dataset of cylinder projections, Fig. 4. In the case of the maximum intensity projections, we generate kymographs illustrating changes over time in the segmented waves projected onto the anteroposterior axis. In the case of the cylinder projections, we show a kymograph along the dorsoventral axis, which depicts the wave segmentations of both sides of the embryos over time.

#### Divergence maps

While the focus of this paper is on segmenting the contraction waves, we briefly explored the concurrence between contraction waves and other vector field descriptors. In addition to the velocity distributions used for the wave segmentation, we computed divergence maps from the PIV results on the 2D cylinder projections. The divergence is useful to identify areas where the tissue density increases (constriction, corresponding to negative divergence) or decreases (expansion, corresponding to positive divergence). Divergence is defined as the sum of partial derivatives of each vector field component. This is usually implemented by computing vector field derivatives between adjacent elements. Computing divergence on immediate neighbors requires the input vector fields to be very smooth, which is usually achieved by averaging the vector fields with a kernel of a certain size. In these cases, the size of the averaging kernel implicitly defines the scale of the divergence. Here, we compute divergence by constructing an expanding template vector field of the desired size and convolving it with each PIV vector field. This expanding template contains normalized vectors pointing away from the center of the template. The size of the template determines the scale of the divergence patterns. We used a kernel size of 17 × 17 pixels to compute the divergence on the PIV results generated from the 2D cylinder projections.

## Results

### Contraction waves coincide with high velocities

By visual examination of long-term live-imaging data of *Tribolium* embryogenesis, we identified contraction waves as rapid back-and-forth movements of cell nuclei with an initial phase of movement in the posteroventral direction, followed by a retraction in the anteroposterior direction. To keep the notions simple, we refer to the first phase as ventral (V), and the second as dorsal (D) phase. We visualized the two-phase motion by overlaying the 2D lateral maximum intensity projections of two consecutive frames in a nuclear-labeled dataset (Fig. 1A).

We used PIV on the lateral maximum intensity projections. Contraction waves manifest in the PIV vector fields as large regions of displacement exhibiting ventral and then dorsal velocity components (Fig. 1B). We examined the temporal changes in PIV vectors at individual positions within contraction waves and found that the V and D phases are associated with locally high velocities. For the temporal resolution used here (3 min), the velocities exhibit a peak during the V phase of the waves (shown exemplarily for one position in Fig. 1C). However, we also observed high velocities for positions outside the contraction region, for instance during rapid retraction of the EE membranes after rupture (Fig. 1C). Therefore, high velocities alone become an unreliable feature for identifying the occurrence, neither in space nor time, of contraction waves after rupture.

### Contraction waves are observed in multiple embryos, different Tribolium lines, and under different microscopy setups

**Fig. 2:**
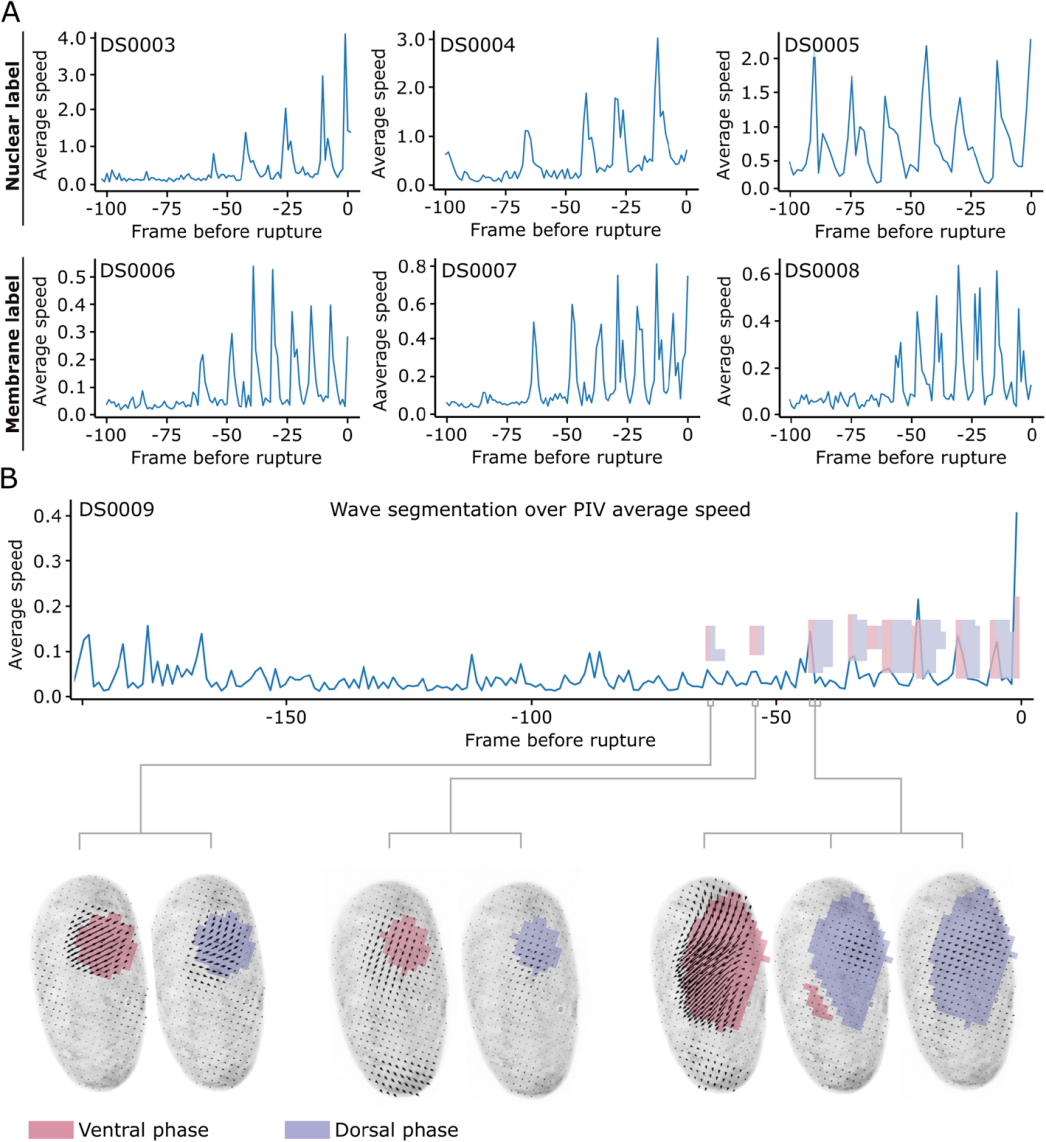
Wave detection from PIV average velocity peaks in multiple embryos. **A** PIV average velocity plots over the 100 timepoints prior to rupture for six different embryos of *Tribolium* exhibiting contraction waves: three of them expressing a nuclear fluorescent markers (DS0003-5) and three embryos expressing membrane-bound fluorescent marker (DS0006-8). PIV results were obtained from 2D+t lateral maximum intensity projections. All embryos display velocity peaks which coincide with contraction waves. The number, relative height and duration of the velocity peaks vary between embryos, manifesting intra-individual variability in the appearance of contraction waves. **B** PIV average velocity plot over the 200 timepoints prior to rupture for one *Tribolium* embryo with membrane labeling (DS0009). This embryo displays noisy velocity peaks prior to the onset of the waves, and two contraction waves do not appear as velocity peaks. The ventral (D) and dorsal (D) phases of these contraction waves were successfully segmented with the proposed algorithm, both in space and time

To demonstrate that the contraction waves are a common phenomenon during *Tribolium* development, we present data on multiple embryos in Fig. 2. In particular, we show results on three embryos with labeled cell nuclei, and four embryos with membrane labeling. We performed PIV on the lateral maximum intensity projections of these datasets and computed the average velocity as a function of time during the 100 frames previous to rupture. We observed that peaks of the average velocity generally coincide with the waves. The velocity peaks in membrane-labeled datasets display lower average speeds, which is partially due to the fact that membrane dynamics introduce a higher degree of signal distortions that reduce the dynamic range of PIV (Fig. 2). These results provide evidence of the consistent presence of contraction waves during the embryonic development of *Tribolium*, as seen from the numerous PIV average velocity peaks in all embryos.

A closer examination of the average velocity peak positions reveals intra-specimen variability in the number, relative height, and duration of the velocity peaks during individual contraction waves. For instance, unlike in Fig. 1C, which displays two distinct peaks for each pair of V and D phases, contraction waves sometimes appear as a single narrow peak in several embryos (e.g. DS0006, DS0007, DS0008 in Fig. 2A). These correspond to waves with short V and/or D phases. Furthermore, one of the investigated embryos with membrane labeling (DS0009) exhibited contraction waves that did not appear as peaks in the average velocity plot (Fig. 2B). When analyzing this dataset with the algorithm described in this paper, which includes directional information to segment the waves, we were able to identify the V and D periods of the faint contraction waves that we observed by eye (see Fig. 2B).

These examples show that the positioning and shape of the velocity peaks is not a reliable marker for identifying individual phases of contraction waves and their durations. The spatial segmentation algorithm provides additional relevant and necessary information to detect and quantify the contraction waves. A more detailed explanation of the segmentation algorithm applied to various representations of a data set from an embryo with a nuclear label is provided in the following section.

### Segmentation of pre-rupture and post-rupture contraction waves from lateral maximum intensity projections

**Fig. 3:**
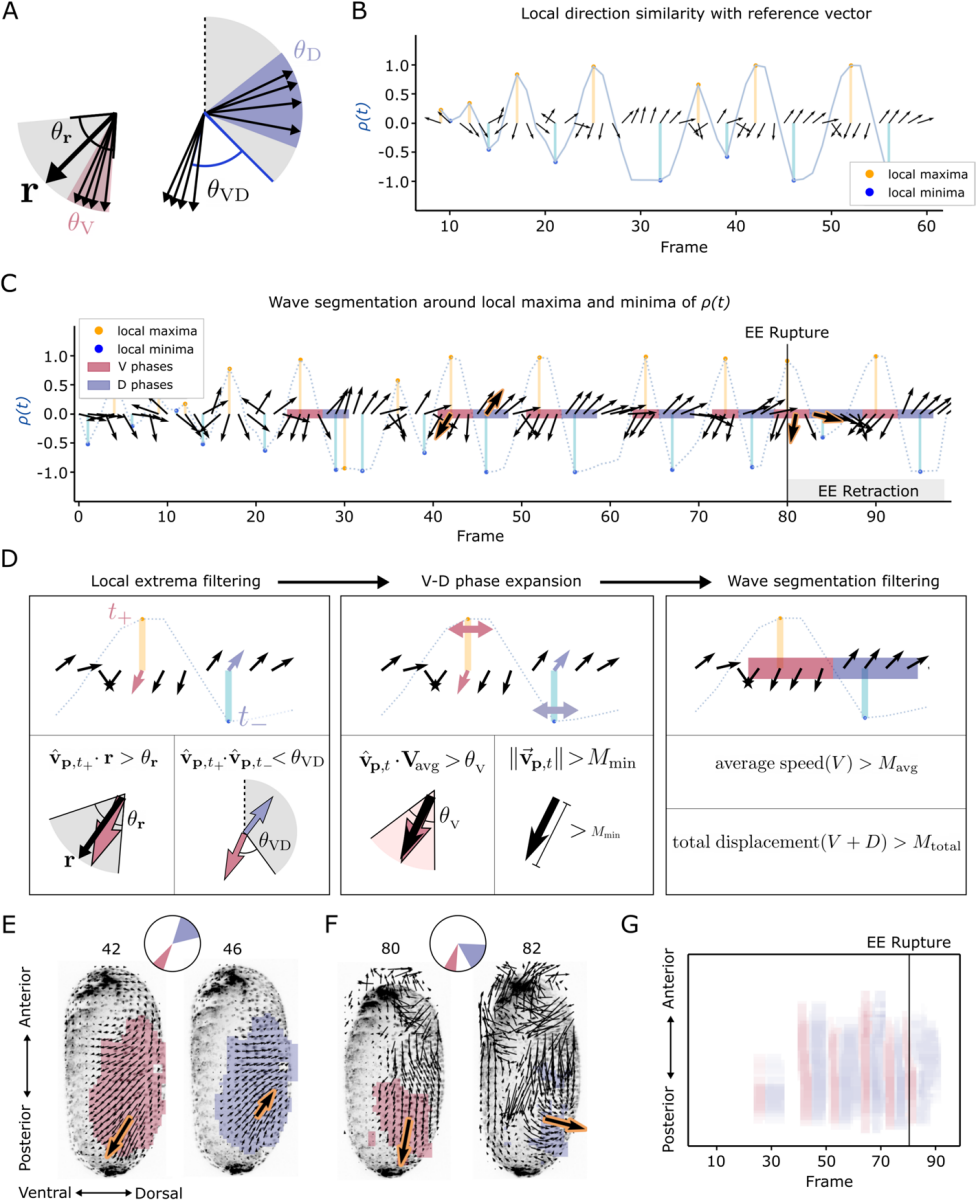
Wave detection algorithm for 2D lateral projections. **A** We define a set of allowable directions for the ventral (V) phases by considering an angular deviation ($\theta _{\textbf{r}}$) around the direction of a reference vector ($\textbf{r}$). The direction of the D phase is restricted to $(-90^{\circ },\;90^{\circ })$ from the dorsal axis, and by the angle to the average direction of the V phase ($\theta _{\textrm{VD}}$). We also limit the angular deviation between the vectors within the V and D phases, $\theta _{\textrm{V}}$ and $\theta _{\textrm{D}}$. **B** Sliding window quantification of direction similarity $\left(\rho (t)\right)$ on a vector time series, ${\textbf{v}}_{\textbf{p},\;{t}}$, between time points 10 and 60. **C** Wave segmentation results for the same vector time series. **D** The segmentations in panel C were generated starting with pairs of local maxima and minima of $\rho (t)$ whose directions are consistent with the a priori angles for the V and D phases (left). The expansions of the V and D segmentations (middle), and their post-processing (left) are parametrized by angle and speed-based criteria. **E** Example of 2D wave segmentation results of a pre-rupture contraction wave. Before rupture, the vectors of the V and D phases point in opposite directions. **F** Example of the 2D wave segmentation of a post-rupture contraction wave. After rupture the angle between the V and D phases is smaller than during pre-rupture waves. **G** Kymograph along the anterior-posterior axes of the wave segmentation results on the 2D+t datasets of maximum projections for all 100 frames. Analysis was performed on DS0001

To better segment contraction waves we focus not on the local velocity peaks but on directional information during changes between movement phase in posteroventral direction followed by a phase of movement in dorsal direction. We designed the algorithm around a set of criteria on the relative changes in movement direction. These criteria define a flexible set of allowable directions for the V and D phases (Fig. 3A). First, we process the PIV vector fields using a sliding window approach (Sect.  ''Temporal segmentation of characteristic dynamics based on PIV vector fields'', Fig. 3B). This step allows us to detect candidate positions for the V and D phases. We then construct the V and D phase segmentations iteratively in a second step. This approach enables us to capture contraction waves of variable duration. As a first simple test case, we generated segmentations of the contraction waves both in time (Fig. 3C) and space (Fig. 3 E–G) for 2D lateral maximum intensity projections of the fused volumes.

We chose a set of parameters that ensures consistent direction of movement in the V and D phases, but allows for a large flexibility in the angle defining the ventral movement, and also in the relative angles of motion between the V and the D phases. With these parameters, the algorithm can detect pre-rupture waves (Fig. 3E) and post-rupture waves (Fig. 3F) with the same set of parameters, even though the differences in directions of movement between the V and D phases in pre- and post-rupture waves is very different. In the kymograph, we identify the presence of five contraction waves before rupture, and one after rupture (Fig. 3G and Additional video 2).

After frame 80 the EE membranes rupture and retract to the dorsal side. This involves the collective movement of the entire EE membranes and high velocities at the retracting edge. The retraction phase might also introduce significant ventral and dorsal movement components. However, the choice of parameters (see Table 1) allows the contraction waves to be distinguished from the dynamics of EE membrane retraction.

### Wave segmentation on the full EE surface illuminates the spatial characteristics of the contraction wave

**Fig. 4:**
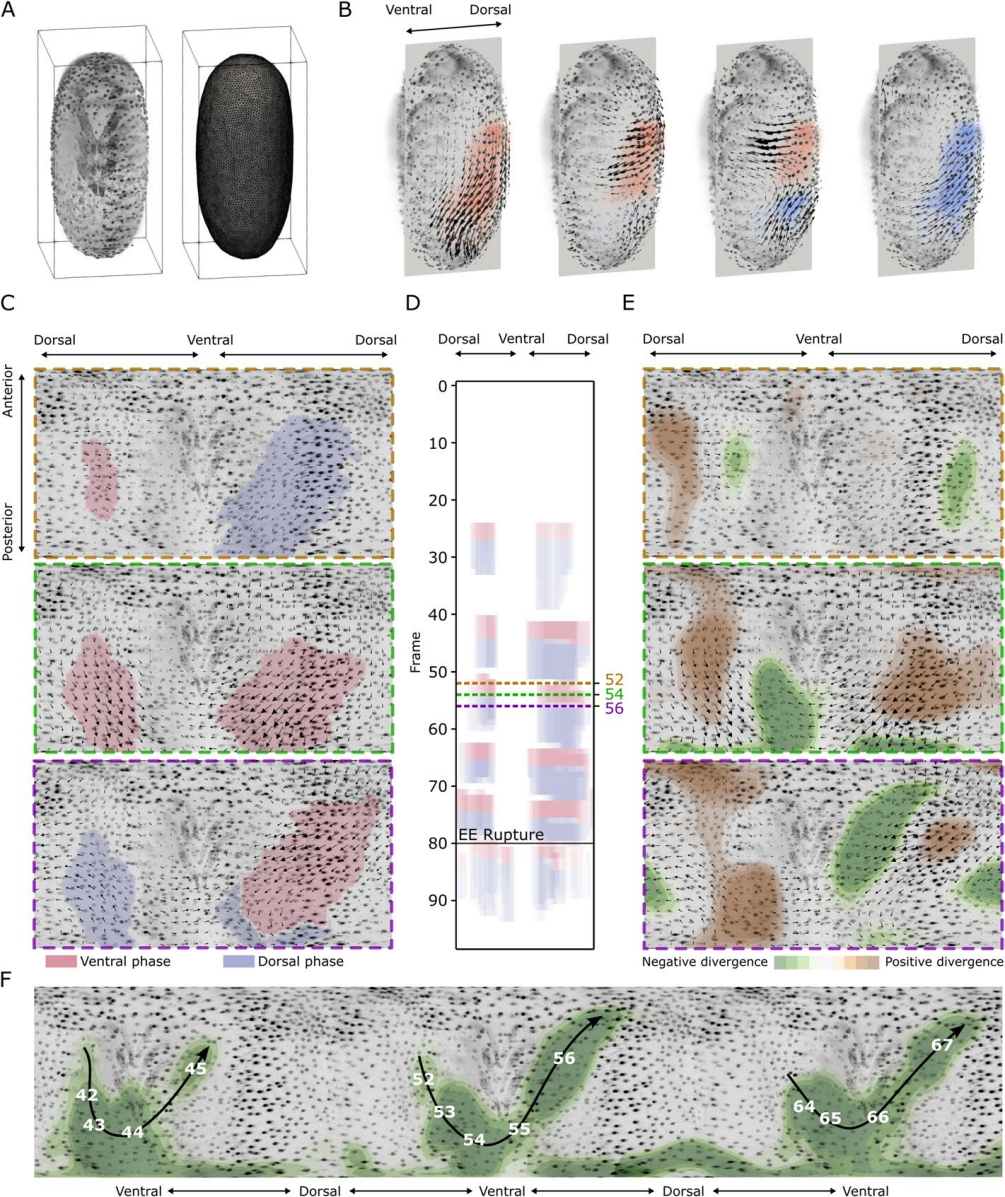
Wave detection in 3D volumes and on 2D cylinder projections. **A** Example of surface mesh extraction for one frame. We used the detected surfaces both as a mask during 3D PIV analyses and to project the tissue surface onto a cylinder. **B** Wave detection on 3D+t vector fields. **C** Wave detection on 2D+t cylinder projections. These projections provide a 360° view of the surface, allowing to detect and visualize contraction waves continuously on both lateral sides of the embryo. The three panels show the progression (from top to bottom) of one contraction wave. The segmentation on the left side is smaller and precedes the one on the right side. **D** The kymograph summarizes the wave detection for all frames, showing the presence of five waves before rupture and one after rupture. **E** In addition, by analyzing the divergence of the PIV vector fields from the cylinder projections, we observed a flow of positive and negative divergence extrema from the left side of the embryo to the right side. This divergence flow correlates with the progression of the wave. **F** Negative divergence (convergence) flows during the three waves prior to rupture. We exploit the periodic boundary of the cylinder projections to visualize the progression of the divergences flow across time by concatenating a projection of frame 54 three times and representing negative divergence calculated from selected frames between 42 and 67. Analysis was performed on DS0001

In the next step, we analyzed the spatial dynamics of the contraction waves in 3D. Because we wanted to focus our analysis only on the EE membranes, we extracted the surface of the embryo from fused 3D image data (Sect.  ''Surface mesh'', Fig. 4A) and used it as a mask for the 3D PIV analysis. The masked data, focused only on the EE membrane regions, offers multiple benefits: (1) the input data for 3D PIV is smaller, making PIV computationally cheaper and faster, and (2) the mask reduces interfering signals, e.g., motion of the embryo proper, which might impact PIV results.

We performed 3D PIV on the masked 3D volumes, and used the same parameters for contraction wave detection and segmentation as for the lateral projections (Sect. ''Lateral maximum intensity projections''). In particular, we used a single static reference vector ${\textbf{r}}$ pointing in the ventral direction. With this approach, the contraction waves are detected and can be segmented and visualized also in 3D (see Fig. 4B and Additional video 5). PIV analysis and subsequent contraction wave segmentation in 3D showed that the contraction waves occur on both lateral sides and are largely synchronized. With the static reference vector, however, we expect to capture only a fraction of the tissue area involved in the contraction dynamics as the movement occurs on a curved surface. Hence, movement towards the ventral area involves a dorsal-ventral direction only in lateral regions, while in ventral and dorsal areas lateral components dominate. A possible solution to better capture the 3D dynamics would be to use a spatially resolved reference vector field tangential to the surface.

Instead, we decided to adopt tissue cartography, a method to represent data from a (closed) surface in 3D by projection onto 2D maps (Fig. 4C). PIV was performed on the 2D cylinder projections, using correction of the length distortions (Sect. ''Cylinder projections''). For wave segmentation we used two reference vectors, ${\textbf{r}_1}$ and ${\textbf{r}_2}$, for the vectors on the right and left side of the embryo. The direct visualization of the contraction waves on the cylinder projections as well as a kymograph along the dorsoventral axis allow for a comparison of the waves on the left and the right side of the embryo (Fig. 4D, Additional Fig. 1 and Additional video 3). We observed that the contraction waves on the left side preceded the waves on the right side (Fig. 4D and Additional Fig. 1). This phase shift was very small (one frame, i.e., 3 min), and is probably a result of the delay in image acquisition between the two lateral sides, which is about one and a half minutes. The tissue area involved in the contraction waves on both lateral sides also differs, with the area on the left being consistently smaller than the segmented area on the right (Fig. 4C). In addition, the kymograph (Fig. 4D) shows that the duration of the V phase is generally shorter than the D phase, a dynamics indicating an abrupt contraction followed by an extended relaxation phase. This consistent signature of the two contraction wave phases is also supported by the velocity dynamics at individual positions, as shown in (Fig. 1C). This analysis was repeated for another dataset of *Tribolium* with a membrane labeling to provide a biological replicate (Additional Fig. 1). Those additional results show the reliability of the wave segmentation pipeline on a dataset with a remarkably different signal (membrane vs nuclear signal) than the first one.

### A repetitive trajectory of divergence minima coincides with the progression of contraction waves

Finally, we used the divergence of the vector field to identify tissue regions in the EE membranes that, by local constriction (negative divergence) and expansion (positive divergence), might generate the observed waves. We quantified the local divergence minima and maxima during the frames of ongoing contraction waves and overlaid them on the cylinder projections (Fig. 4E). Here, we observed a characteristic pattern in the spatial localization of the divergence minima for several consecutive time frames (Fig. 4E). Specifically, the minima moved from one lateral side of the embryo proper, down to the posterior pole, and up to the other lateral side, aligning with the progression of a contraction wave. This pattern repeats for each detected wave in a similar manner, as shown by augmenting cylinder projections (Fig. 4F) and in Additional video 4.

## Discussion

We present a computational approach for the analysis of movement patterns in 3D biological tissues that involves quantification of displacements by PIV, segmentation of tissue areas showing characteristic back-and-forth movement and different representations of 3D microscopy data. Here, we applied it to detect contraction waves in the EE membranes of the red flour beetle *Tribolium castaneum*. Central to our approach is the algorithm for the detection and segmentation of alternating movements in two directions in 2D+t and 3D+t biological datasets.

This algorithm is based on selecting a flexible set of rules to detect a priori defined patterns. These rules combine angle-based and speed-based criteria, since velocities alone are not enough to differentiate between EE contraction waves and other rapid developmental processes, e.g., EE membrane rupture and retraction. The algorithm includes only a few parameters to control the components of the two-phase movements and the tolerances. Here, the tolerances are chosen flexibly, which is important because pre- and post-rupture contraction waves have different directions of movement and different relative angles between the two phases. The fact that the algorithm accepts a separate reference vector for each position of the vector field opens interesting opportunities in the future, e.g. the possibility to define multiple locations around which wave-like movement patterns may be detected.

We applied the wave segmentation approach separately to three different representations of multiple *Tribolium* datasets: (1) lateral maximum intensity projections, (2) 3D fused data, and (3) cylinder maximum intensity projections. For each of these representations, we defined a set of reference vectors indicating the wave direction we want to segment. Wave segmentation on the lateral maximum intensity projections requires only one reference vector, and it already allowed us to quantify a change in the contraction wave orientation after EE membranes rupture. To study the full spatial characteristics of the contraction wave, we proceeded to analyze fused 3D data. In a 3D representation of the data, however, we found that using only one reference vector is not sufficient to segment the full area of a wave due to local tissue curvature. Here, each vector field position would require a unique reference vector tangential to the surface. Instead of expanding to a field of reference vectors, we created cylinder projections on which we could define two mirrored reference vectors and corrected our wave segmentation for distortions [30]. The advantage of this approach is that we unroll the EE membranes into a flat cartographic map allowing for 2D analysis of the surface. This approach also benefits from the reduction of potential signal contributions from the embryo proper and the yolk.

Additionally, cylinder projections enabled us to track a "U"-shaped flow of negative divergence in consecutive frames, which was characteristic of multiple waves: the contraction propagates posteriorly over one side of the embryo, crosses the ventral line, and moves anteriorly over the other side. This might be indicative of a propagating focus of contractility that tugs on the neighboring tissue and causes the displacement of nuclei, which we perceive as a contraction wave. The trajectory of this contractile focus is non-intuitive, and might hint at a more complex interaction between the geometry, as well as structural and regulatory elements involved in the contraction wave dynamics in this organism. In any case, the repetitive unidirectional shift of the divergence minima may be an interesting biological discovery and needs to be supported by a bigger sample size.

The workflow proposed here was motivated by the discovery of rapid and repeated contraction waves in *Tribolium* that occur before serosa rupture. Although contractile behavior is known to occur in EE membranes of other insect species [15, 44], the contractions have not been shown before to be organized in a wave-like fashion, potentially due to technical difficulties in recording these fast movements with the necessary temporal resolution. The data recorded and presented in this paper allows for a quantitative analysis to characterize the contraction waves in individual specimens, regarding the number, frequency, temporal regularity, direction of motion and its dynamics, duration of the respective phases, the size of the involved membrane area, or the symmetry between the two lateral sides. The aim of this article is to provide detail on the technical approach for the quantitative analysis, which provides the foundation for future studies on the variability of these quantities in different embryos of the same line, the comparison between different lines, and the dependence on factors such as temperature or the senescence of the parent. We also expect the proposed analysis workflow to be useful in the future, e.g., when similar contraction waves are discovered in further species, but also for the analysis of tissue dynamics showing akin behavior not related to development. Regarding the quantitative analysis of the contraction waves, already in the small sample presented here, we have learned that the contraction waves occur repeatedly with a roughly constant frequency, continue after rupture and during the retraction phase with a similar frequency but different angle, exhibit a characteristic local velocity signature, a short ventral and extended dorsal phase, as well as symmetry between the two lateral sides. To characterize the phenomenon further, we currently investigate the variability of the spatiotemporal dynamics of the contraction waves in a larger number of samples and under various conditions.

## Conclusions

The computational workflow presented here was developed to automate the detection of previously undescribed contraction waves of EE tissues during the development of Tribolium castaneum. While these waves were discovered through visual examination of the data, it was necessary to establish a robust computational pipeline to replace the manual segmentation of the waves. In this way, we overcome the need for human intervention, which is remarkably time-consuming for time-lapse three-dimensional recordings, inherently subjective, and prone to variability, particularly when analyzing subtle, irregular, or noisy patterns. In contrast, our workflow detects contraction waves from robust PIV results and generates repeatable quantitative measurements for each detected wave. For example, our pipeline quantifies the movement speed, duration, and magnitude of each wave while deriving secondary features like divergence maps–metrics that can not be obtained through visual analysis. Additionally, the presented pipeline is capable of detecting subtle movement patterns that might otherwise go unnoticed by visual inspection.

Having established the reliability of the presented pipeline, we will deploy it in our future work to not only qualitatively characterize and segment the contraction waves but to statistically evaluate the number, size, and shape of contraction waves in multiple embryos. With this, we will answer questions revolving around contraction wave dynamics: e.g., How many waves occur before and after rupture? Does the area of the waves differ between consecutive wave cycles? Do they change their shape, orientation, or location?

In addition, the short computing time and robustness of the workflow, especially on 2D lateral projections, make it also relevant for smart microscopy where, e.g., a faster acquisition mode is triggered by characteristic spatiotemporal patterns [45]. With a dedicated smart microscopy setup it will be possible to achieve the necessary high temporal resolution for the analysis of rapid dynamics, such as rupture and retraction of EE membranes, while keeping overall light exposure on the sample as well as data size at a reduced level.

Last but not least, the wave detection presented in this work is applicable or could be adapted to segment movements in other biological systems, e.g., rhythmic yolk contractions in goldfish eggs and embryos [46], or fast contraction waves in the simple marine animal *Trichoplax adhaerens* [3]. The only condition of our pipelines is for the spatiotemporal patterns of interest to be collective and quantifiable by PIV, and that the desired pattern can be expressed as a local back-and-forth movement. By providing a standardized, computationally robust methodology, our approach not only enhances existing morphogenetic research techniques but also contributes to a more comprehensive and systematic understanding of developmental processes.

## Additional file

Additional file 1: Projections of the 3D+t dataset of *Tribolium castaneum*. This video shows maximum intensity depth-adjusted projections from 4 perspectives (ventral, left lateral, dorsal, right lateral) extracted from the multiview reconstructed 3D+t recordings of a *Tribolium* embryo displaying nuclear fluorescent labeling. The movie contains 100 frames, covering 5 h of the late development of *Tribolium* with a time interval of 3 min. Contraction waves start at the anterodorsal region and propagate along the lateral sides in the posteroventral direction. The first contraction wave happens at timestamp 01:21. Rupture of EE membranes happens at timestamp 04:06.

Additional file 2: 2D wave segmentation for lateral maximum intensity projections. The movie shows 2D+t wave segmentation results obtained by analyzing 2D+t lateral projections from the left lateral side of a 3D+t dataset of the late development of a *Tribolium* embryo. The ventral phases of the segmented contraction waves are highlighted in red, and the dorsal phases in blue. We observe and detect four contraction waves before rupture, and one after rupture.

Additional file 3: Wave segmentation on cylinder projections in a membrane-labeled embryo. This figure shows wave segmentation results on cylinder projections computed from a membrane-labeled *Tribolium* embryo. These results are akin to those shown in Fig. 4. Namely, this embryo displays contraction waves on both lateral sides of the embryo, with a small delay between the onset of the waves between the two sides. In addition, we also observe 5-6 clearly defined waves before rupture, followed by 4–5 post-rupture waves in quick succession. Analysis was performed on DS0002. Supplementary file 3 (png 2492 KB)

Additional file 4: 2D wave segmentation for cylinder maps. This video shows 2D+t wave segmentation results obtained by analyzing 2D+t cylinder projections extracted from a 3D+t dataset of the late development of a *Tribolium* embryo. These projections provide a 360° view of the surface of the embryo, which contains predominantly signal from the amnion and serosa EE membranes. The ventral side occupies the middle of the projections. The ventral phases of the segmented contraction waves are highlighted in red, and the dorsal phases in blue. We detect contraction waves almost simultaneously on both lateral sides of the embryo.

Additional file 5: Divergence map for cylinder maps. This video depicts divergence maps computed from 2D PIV vector fields of the 2D+t dataset for cylinder projections extracted from a 3D+t dataset of the late development of a *Tribolium* embryo. These projections provide a 360° view of the surface of the embryo, which contains predominantly signal from the amnion and serosa EE membranes. The ventral side occupies the middle of the projections. Patterns of contraction (negative divergence) and expansion (positive divergence) are visualized in green and orange, respectively. During contraction waves, divergence minima (green signal) appear to follow a "U"-like trajectory: propagating from the left to the right sides of the video, crossing the ventral line over the posterior end of the embryo.

Additional file 6: 3D wave segmentation for 3D reconstructed volumes. This video shows 3D+t wave segmentation results computed for the 3D+t dataset of fused volumes of a *Tribolium* embryo. The ventral phases of the segmented contraction waves are highlighted in red, and the dorsal phases in blue. The camera rotates around the anteroposterior axis of the embryo to visualize the wave segmentation results from all sides of the embryo.

## Data Availability

All datasets analyzed in this paper have been uploaded online and are available through the following Zenodo DOI: 10.5281/zenodo.13744448. This includes pre-computed results (such as surface meshes or distortion maps), which can be easily loaded to reproduce the results. The source code of our wave segmentation algorithm, as well as jupyter notebooks to reproduce the results are available from Github: https://github.com/Marc-3d/contraction_wave_segmentation.
