# Supplementary figures and images for: Spatiotemporal segmentation of contraction waves in the extra-embryonic membranes of the red flour beetle
Source: BMC Bioinformatics. 2025 Oct 21;26:253. doi: 10.1186/s12859-025-06259-1 (PMC12538966; doi:10.1186/s12859-025-06259-1)

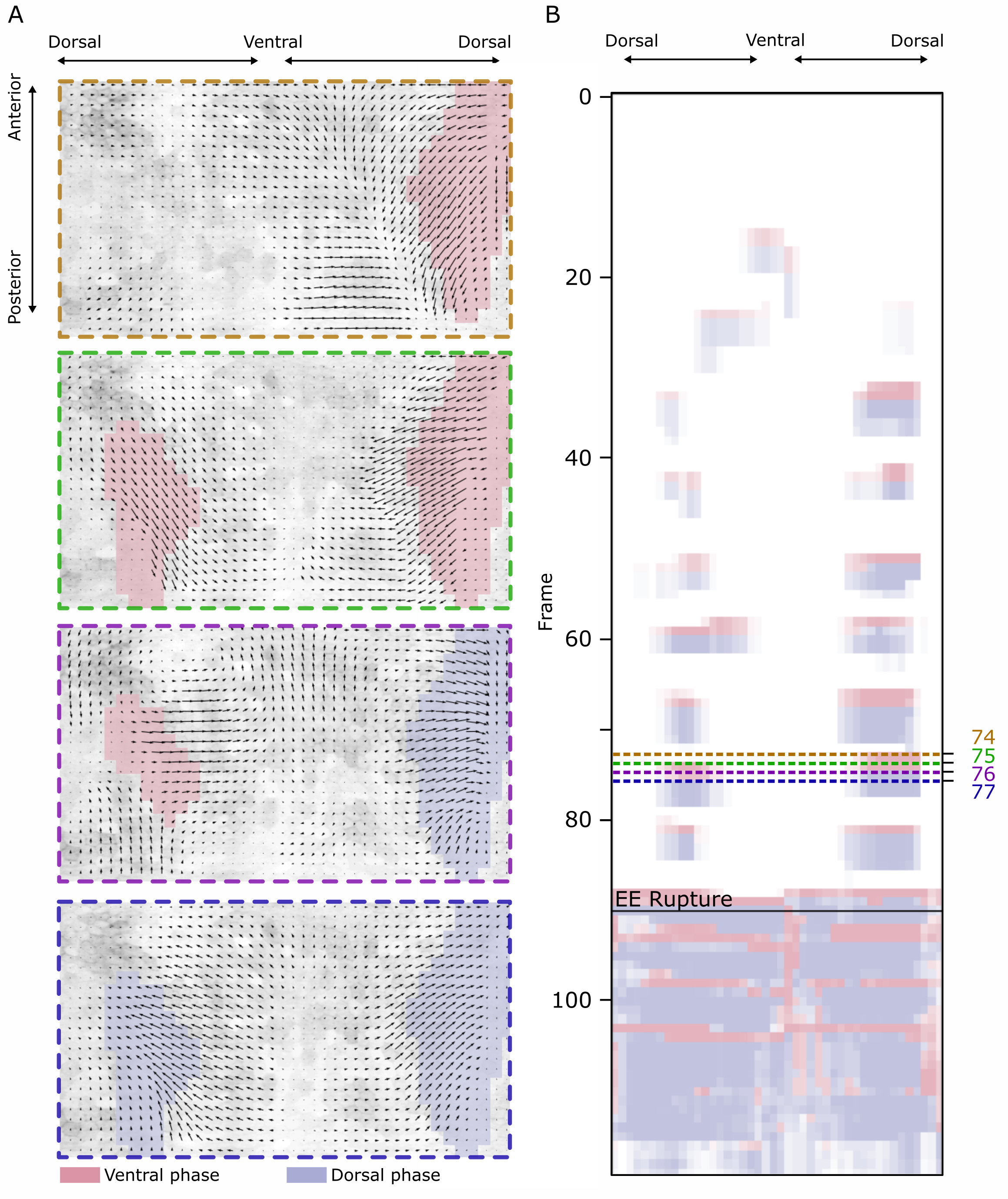

Supplement: Supplementary file 3 — Additional file 3: Wave segmentation on cylinder projections in a membrane-labeled embryo. This figure shows wave segmentation results on cylinder projections computed from a membrane-labeled Tribolium embryo. These results are akin to those shown in Fig. 4. Namely, this embryo displays contraction waves on both lateral sides of the embryo, with a small delay between the onset of the waves between the two sides. In addition, we also observe 5-6 clearly defined waves before rupture, followed by 4–5 post-rupture waves in quick succession. Analysis was performed on DS0002. [file 12859_2025_6259_MOESM3_ESM.png]
